# Surface Phosphatidylserine Is Responsible for the Internalization on Microvesicles Derived from Hypoxia-Induced Human Bone Marrow Mesenchymal Stem Cells into Human Endothelial Cells

**DOI:** 10.1371/journal.pone.0147360

**Published:** 2016-01-25

**Authors:** Xiaojuan Wei, Chaozhong Liu, Hengxiang Wang, Lisheng Wang, Fengjun Xiao, Zikuan Guo, Hongchao Zhang

**Affiliations:** 1 Department of Cardiology Surgery, General Hospital of Air Force, Beijing, China; 2 Department of Hematology, General Hospital of Air Force, Beijing, China; 3 Department of Experimental Hematology, Beijing Institute of Radiation Medicine, Beijing, China; University of Torino, ITALY

## Abstract

**Background:**

Previous data have proven that microvesicles derived from hypoxia-induced mesenchymal stem cells (MSC-MVs) can be internalized into endothelial cells, enhancing their proliferation and vessel structure formation and promoting *in vivo* angiogenesis. However, there is a paucity of information about how the MSC-MVs are up-taken by endothelial cells.

**Methods:**

MVs were prepared from the supernatants of human bone marrow MSCs that had been exposed to a hypoxic and/or serum-deprivation condition. The incorporation of hypoxia-induced MSC-MVs into human umbilical cord endothelial cells (HUVECs) was observed by flow cytometry and confocal microscopy in the presence or absence of recombinant human Annexin-V (Anx-V) and antibodies against human CD29 and CD44. Further, small interfering RNA (siRNA) targeted at Anx-V and PSR was delivered into HUVECs, or HUVECs were treated with a monoclonal antibody against phosphatidylserine receptor (PSR) and the cellular internalization of MVs was re-assessed.

**Results:**

The addition of exogenous Anx-V could inhibit the uptake of MVs isolated from hypoxia-induced stem cells by HUVECs in a dose- and time-dependent manner, while the anti-CD29 and CD44 antibodies had no effect on the internalization process. The suppression was neither observed in Anx-V siRNA-transfected HUVECs, however, addition of anti-PSR antibody and PSR siRNA-transfected HUVECs greatly blocked the incorporation of MVs isolated from hypoxia-induced stem cells into HUVECs.

**Conclusion:**

PS on the MVs isolated from hypoxia-induced stem cells is the critical molecule in the uptake by HUVECs.

## Introduction

Cardiovascular and peripheral blood vessel diseases are the commonest conditions in the elderly. Usually, atherosclerosis is the underlying disease which is initiated and aggravated by the continuous defects of integrity in the vascular endothelium, resulting in the vessel occlusion and subsequent damage and dysfunction of the involved tissues and organs. Mesenchymal stem cells (MSCs) are adult stem cells characterized by their immuno-regulatory, hematopoiesis-supporting and angiogenesis-promoting activities. According to the reports, many tissues have been demonstrated to be isolated the MSCs, including bone marrow, adipose tissue, liver, muscle, amniotic fluid, placenta, umbilical cord blood, umbilical cord and dental pulp[1]. In the clinical, bone marrow is more conveniently obtained. In addition, it has lower immunogenicity, and can obtain more stem cells. At present, MSCs are the prominently promising stem cells in the design of novel therapeutic intervention in both cardiac and peripheral blood vessel diseases [1–4].

Increasing clinical trials have been performed to testify the safety and effectiveness of MSCs in the management of these ischemic diseases [5–8]. However, some investigators have raised doubts about the safety of MSC application [9] and the mechanisms of MSC therapy are still in dispute [3, 10].

Interestingly, the transplanted MSCs will be exposed to the microenvirment of hypoxic and ischemic in these diseases. Previous study demonstrated that MSCs are able release large quantities of microvesicles (MVs) under a hypoxic and/or serum-deprivation condition [11]. MVs from hypoxia-induced MSCs (MSC-MVs) can be internalized into endothelial cells, enhancing their *in vitro* proliferation and vessel structure formation and promoting *in vivo* angiogenesis as well [11]. The angiogenesis-promoting activity of MSC-MVs has been further identified by other investigators, using a rat myocardial infarction model [12] and a mouse subcutaneous blood vessel formation model [13]. Meanwhile, it should be noted that the internalization of MSC-MVs into endothelial cells is the first and determinant process that gives rise to the transfer of bioactive molecules encapsulated in the vesicles into the host cells. However, the mechanisms underlying the internalization remain still elusive.

Recent studies indicate the microvesicles was a vital mediator in the cell-to-cell communication, and internalization may be the key process. Accordingly, numerous researchers showed the contents of MVs vary greatly depending on the originate cells, nevertheless, all the MVs contain some endogenous substances including membrance traffic proteins (i.e.RabGT-Pases, annexins, flotilin), multivesicular bodies (i.e.TSG101, Alix), intergins and tetraspanins (CD9, CD63, CD81,CD29). Additionally, the raft-lipids (cholesterol, flotillins) and some signal transduction (EGFR, PI3K) also have been detected[14,15]. Katrin J. Svensson’s research have showed the uptake of MVs may through the raft-lipids mediated endocytosis[16]. In other studies, membrance fusion and trafficking proteins interactions were certified to the pathway of internalization[17,18]. Moreover, some researchers found that phosphatidyl-serine (PS) plays a vital role in uptaking signal and the effect of exosomes application on some target cells growth[19].

In the present study, the potential pathway that hypoxia-induced MSC-MVs enter into human umbilical cord endothelial cells (HUVECs) has been probed and, the results here suggest that the interaction of phosphatidylserine (PS) on the MVs isolated from hypoxia-induced stem cells, with the PS receptor (PSR) on the HUVECs is largely responsible for the incorporation.

## Materials and Methods

### Cell culture

In this study, all the Human Bone marrow samples and umbilical cords were collected after an informed consent was given, and in accordance with the Ethics Guidelines for Research Involving Human Subjects or Human Tissue from the General Hospital of Air Force. All procedures have been reviewed and approved by the Institutional Review Board (IRB) of Academy of Military Medical Sciences. All the participants provide their written informed consent to participate in the study. In the subject application stage, all the participants had learned the research content carefully, and provided the informed consents. And the related organization of Air Force General Hospital checked the informed consents, then the National Natural Science Foundation approved. The ethics committees/IRBs approve this consent procedure.

The BMSCs were isolated and identified as previously described [20]. Briefly, the mono-nucleated cells were isolated by density gradient centrifugation and differential adhesion method was used to isolate BMSCs. Then the cells cultured in a serum-free and animal component free medium according to the protocol described by the manufacturer (Beijing Sanley Bio-tech Co, Beijing, China) at 37°C,5%CO_2_. The fresh medium was changed every 2–3 days, and the cells were sub-cultured at 90% confluence.

HUVECs were isolated and cultured according to the protocol of our lab [11]. In brief, the umbilical cord vein was collected and syringed twice with Hanks Balanced Salt Solutions, then 0.25% trypsin was filled into the vein and reacted at room temperature for 30min. Subsequently, PBS was used to wash the vein and collected to centrifuge to isolate the cells. Then the cells were seeded onto a six-wells plate, which was pre-coated with PBS containing 2% gelatin and fibronectin (5μg/ml) at 37°C for 1 hour. The cells were cultured in low-glucose DMEM medium supplemented with 10ng/ml VEGF, 50ng/ml bFGF, 50μg/ml VC, and 2% FBS. And the cells at passage 2 to 3 were used in the experiments described below.

### MSC-MV isolation

MSC-MVs were harvested as previously reported with a mild modification [11]. Briefly, human bone marrow MSCs were allowed to attach overnight, and then the culture medium was removed and changed to fresh alpha-MEM without serum supplement. The cells were then exposed to a hypoxic condition (1%O_2_, 5%CO_2_, 94%N_2_) or normoxia for 72 hours and digested with 0.05% enzyme. Then the cells were washed twice with PBS, and trypan blue exclusion test was used to detect the cells viability. It found that the cells viability was >95%. Subsequently, the supernatants were collected and centrifuged at 1500×g for 15 minutes to remove the cell debris, followed by a filtration through a 0.22μm Super Membrane (Pall Life Sciences). The supernatants were then ultracentrifuged at 170,000×g (Beckman Coulter Optima L-100 XP Ultracentrifuge) for 5 hours at 4°C. The precipitate pellets were then washed twice in PBS and suspended in the apop buffer containing 5mM KCl, 1mM MgCl_2_, and 136mM NaCl. In selected experiments, isolated MVs were resuspended in 1ml PBS that mixed with 5μl DiI or 1μl CFSE (Sigma, USA), and incubated for 20 minutes in dark at room temperature. The labeling reaction was stopped by adding an equal volume PBS of 1% BSA. Then labeled microvesicles were washed twice with PBS and ultracentrifuged at 170,000×g for 5 hours at 4°C. The protein concentration of MVs was determined with the BCA protein assay kit (APPLYGEN, Beijing, China) and accordingly, MVs were split into aliquots and stored at -80°C.

### Identification of MSC-MVs

The identification of MSC-MVs was performed as previously described [11]. MVs were fixed with 3% phosphotungstic acid, laid on copper mesh formvar grids, and examined under scanning electron microscope (Hitachi H-7650). The surface molecule profile was evaluated with the bead-based flow cytometry technique [11]. Briefly, MSC-MVs were bound to aldehyde/sulfate latex beads (4μm; Molecular Probes; Invitrogen) and reacted with FITC-conjugated Anx-V (BD, USA), FITC- or PE-conjugated mouse anti-human CD29, CD31, CD44, CD45 and CD73 monoclonal antibodies that are generally used to identify human MSCs [21]. Meanwhile, specific markers to identify MVs including CD9, CD63 and CD81 were also detected routinely. All the antibodies were purchased from BD, USA.

To further clarify the components of the vesicles, western blot was also performed. Firstly, The MVs and hypoxia-induced MSCs were respectively collected and lysed with 100μl RIPA buffer (Applygen Technologies, China) supplement with 1μl protease inhibitor on ice for 30min. Then pyrolysis products of the cells were centrifuged at 15,000×g for 15min at 4°C and BCA protein assay kit was used to detect the protein concentration. 30μg protein was electrophoresed on 10% SDS-polyacrylamide gel, and transferred to a nitrocellulose membranes(Applygen Technologies, China) at 60 mA for 3 h. The membrane was subsequently washed and blocked with 5% skim milk, and incubated with diluted corresponding antibody (β-actin, CD9, CD63 and D81, cat # A1978, C9993, HPA010088, SAB3500454, Sigma,USA) overnight at 4°C. Lastly, the HRP conjugated anti-rabbit IgG was used and exposed with an X-ray film.

### Confocal laser scanning microscopy

To observe the internalization process of MSC-MVs, HUVECs were seeded into glass-bottomed culture dishes specially designed for confocal observation in the presence of 10μg/ml DiI-labeled MVs and the culture was incubated for the indicated time-points. Unbound MVs were removed by extensive washing with PBS, followed by the cell fixation with 4% Paraformaldehyde for 15min at room temperature and pretreatment with 0.5% Triton-X100 for 10 minutes. The cells were then washed with 5% BSA in PBS with Tween 20 and incubated for 20min to block unspecific binding sites. A rabbit anti-human alpha Tublin antibody (Gene Tex, USA) at a dilution of 1:200 in PBS containing 0.5% BSA was added and the cells were incubated overnight at 4°C. After washing in PBS, a goat anti-rabbit IgG antibody conjugated FITC (Southernbiotech, USA) was added and incubated for 60 minutes at room temperature. The nuclei were counter-stained with 1μmol/L 4',6-diamidino-2-phenylindole (DAPI, Sigma, USA) for five minutes. The cells were observed with a Zeiss LSM 510 Confocal laser scanning microscope, and the images were analyzed by using the Zeiss LSM and volocity Demo software (PerkinElmer, USA). The relative fluorescence intensity of DiI dye was assessed with a software Image-Pro Plus (Media Cybernetics, USA).

Confocal microscopy was also used to detect PSR expression on HUVECs. Briefly, the cells were fixed and reacted with a rabbit monoclonal antibody against PSR (Abcam, USA) at a dilution of 1:200. The cells were washed in PBS and reacted with a FITC-conjugated secondary antibody. Then the cells were observed as described above.

### MV internalization assessed by flow cytometry

HUVECs were cultured in the presence of CFSE-labeled MSC-MVs that had been pretreated with mouse-originated monoclonal antibodies against human CD29 and CD44 (BD, USA) at a dose of 1μg/ml and/or recombinant human Anx-V (Biovision, USA) at graded concentrations. The cells were collected by trypsin digestion and washed twice in PBS. At least 10,000 events were harvested with a flow cytometer (FACS-Calibur, BD). The data were analyzed with the WinMDI 2.9 software after the target events were gated according to the forward and side scatter corner signals. HUVEC co-cultured with unlabled MVs were served as control.

### Tube formation assay

Aliquots of 3×10^4^ HUVECs in a volume of 100μl were seeded into a 48-well plate, which was pre-coated with 100μl matrigel for 1 hour. The cells were then incubated at 37°C, 5% CO_2_ for 24 hours and tube formation was observed by microscopy. Further, to observe the involvement of PS in the incorporation of MVs into HUVECs, MVs (100μg/ml) were mixed with recombinant human Anx-V (10μg/ml) (Biovision, USA) and the reaction lasted for 1 hour at 37°C. Then the mixture was washed with apop buffer and then added into the culture and the tube formation was reassessed. Moreover, the human Anx-V mixed with HUVECs for 1 hour at 37°C, then the cells was washed twice and planted on 48-well plate that including martigel as the negative control. The number of the network structure was quantified by randomly selecting 5 field per well.

### Transfection of small interfering RNA

Small interfering RNAs (siRNA) targeted at human Anx-V and the control siRNA were purchased from Santa Cruz Biotech (Cat No. sc-36324 & sc-37007). The siRNA targeted against human Anx-V and the non-silencing control siRNA were designed according to the sequences reported previously [22]. Transfection was performed with the siRNA Transfection Reagent according to the instructions of the manufacturer (Santa Cruz Biotech). The expression of Anx-V on the surface of HUVECs was detected by flow cytometry after the cells were reacted with an FITC-conjugated anti-human Anx-V monoclonal antibody (eBioscience, USA). Moreover, the PSR-siRNA(sc-36325,Santa Cruz Biotech) and the negative-siRNA (sc-37007,Santa Cruz Biotech) transfections also performed in the HUVECs according to the instructions of the manufacturer (Santa Cruz Biotech). And the expression of PSR on surface of the HUVECs was detected by flow cytometry.

### Statistical analysis

All values are expressed as the mean ± S.D. Statistical analysis was completed with student’s two-tailed unpaired t-test or one-way ANOVA using Graph pad Prism 5. A P-value of <0.05 was considered statistically significant.

## Results

### Identification of MSC-MVs

**Fig 1:**
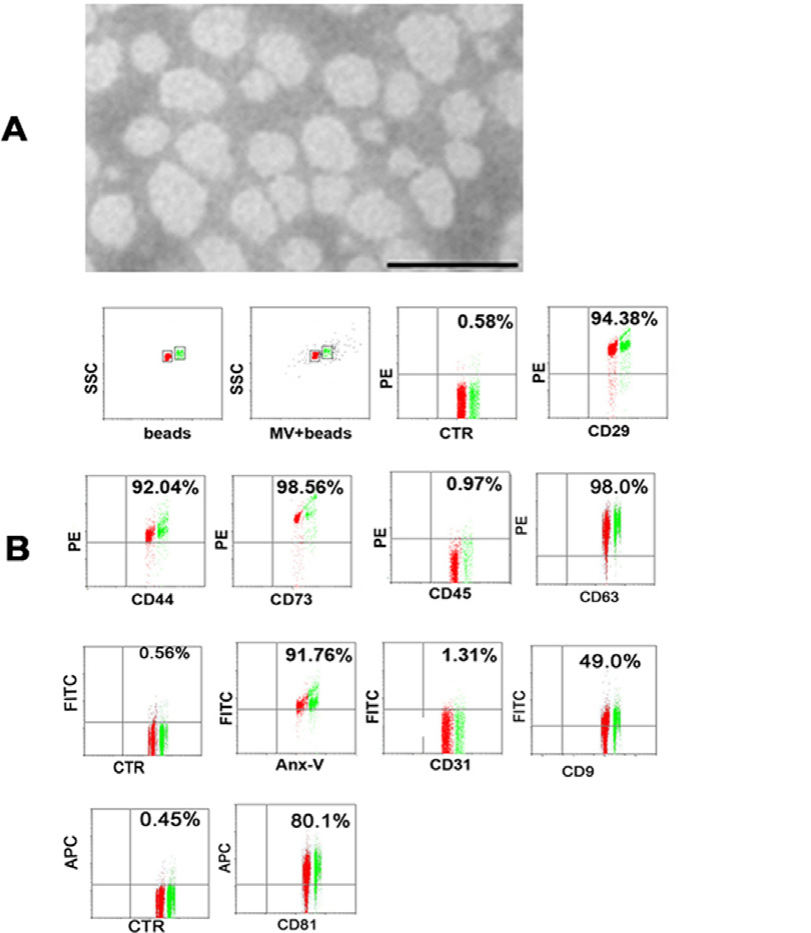
Identification of MSC-MVs with electron microscopy (A) and flow cytometry (B). (A) The bar represents 100nm. (B) MSC-MVs were conjugated with aldehyde/sulfate latex beads and reacted with fluorescein-labeled antibodies or Anx-V. The events were collected with a flow cytometer and the single beads (red) and the doublets of beads (green) were gated for further analysis. The percentages of the positivity in contrast to an isotype antibody are indicated. X-axis: forward scatter corner signals showing the size of the gated events. Beads: Beads were collected for the determination of the gates. Beads+MVs: MVs conjugated with beads were collected for further determination of the gates for analysis. CTR: MVs reacted with a PE-labeled isotype antibody. The results are representative of three individual experiments.

**Fig 2:**
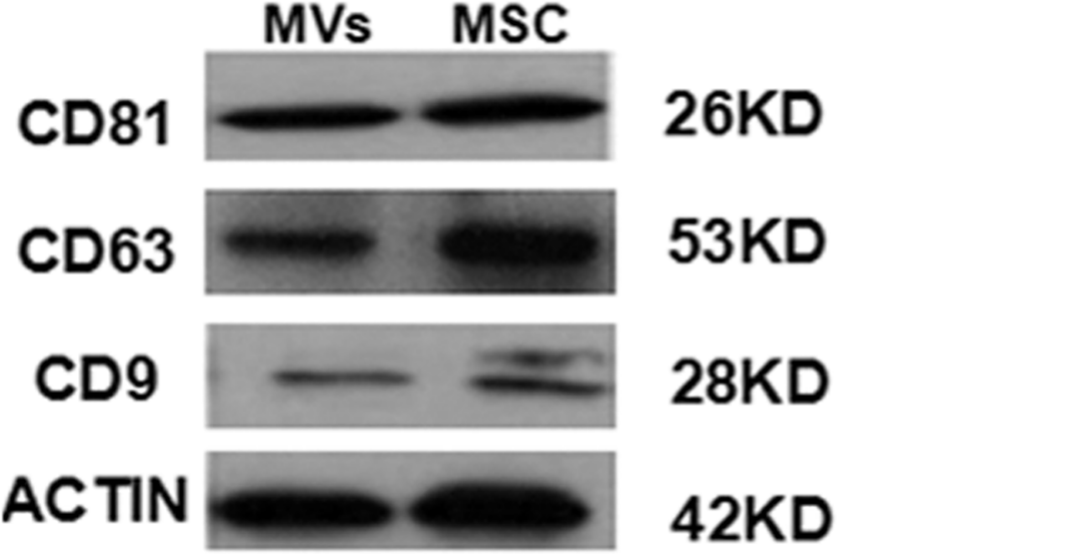
Western blot analysis on the expression of the exosomal markers CD63, CD81, and CD9 in MSCs and exosome-like vesicles.

MVs derived from hypoxia-induced hBMSCs were collected by ultracentrifugation and identified by electron microscopy and flow cytometry. The results showed that the MSC-MVs were relatively homogenous in morphology, round- or cup-shaped with a diameter around 50–100nm (Fig 1A). Flow cytometry revealed that hypoxia-induced MSC-MVs reacted with Anx-V, suggesting PS exposure on their surface (Fig 1B). Similar to the generally accepted surface profile of human MSCs [20], the MVs expressed CD44, CD73, while they were negative for CD31 and CD45. And the specific molecules of MVs such as CD9, CD63, CD81 and the intergins CD29 were also detected (Fig 1B). These proteins were generally accepted on MVs and may include in the uptake process[14,15]. The results revealed that the MVs were originated from hypoxia-induced MSCs and in accordance with the basic characteristics of microvesiciles. About the iconic proteins, the corresponding western blot experiment was also performed. The results showed that the obtained microvesicles expressed CD9, CD81, and CD63, which were in consistent with those from flow cytometric analysis (Fig 2).

According to above methods, the specific molecules were detected after MSCs had experienced serum-free and normoxic condition. Flow cytometry revealed that the MVs derived from the normoxia cells seemed not to react with Anx-V, suggesting no PS exposure on their surface (S1 Fig).

### Time- and dose-dependence of the internalization of MSC-MVs

**Fig 3:**
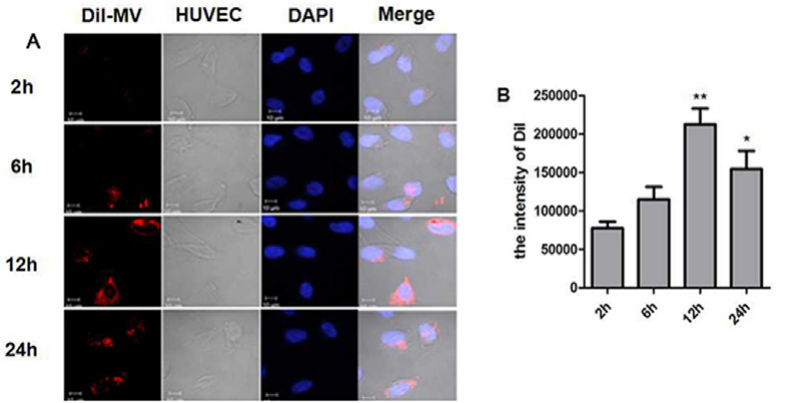
Incorporation of MSC-MVs into HUVECs observed with confocal laser microscopy. (A) DiI-labeled MSC-MVs were added into the HUVEC culture. The cells were fixed and DAPI-stained, followed by confocal observation. Red: DiI-MVs; Blue: DAPI; Ordinary light: HUVEC; and Merge: the merged images of the three above. Bar: 10μm. (B) The mean fluorescence intensity of DiI in per field. The results are representative of three individual experiments.

**Fig 4:**
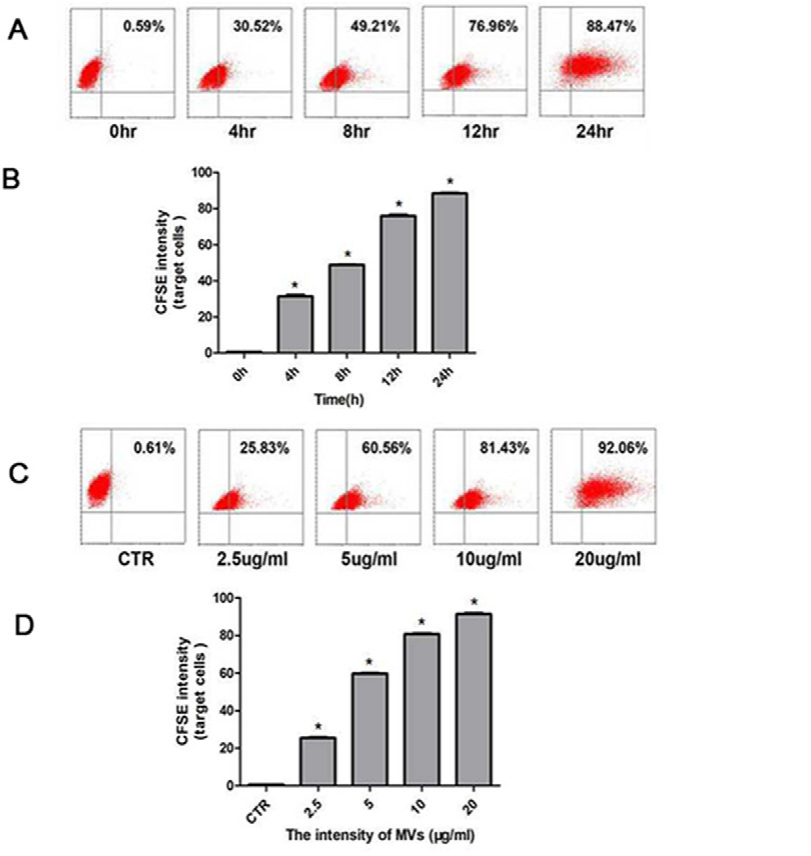
MSC-MVs were engulfed by HUVECs dose- and time-dependent. (A) CFSE-labeled MSC-MVs were added to the culture medium of HUVECs at a dose of 10μg/ml and the cells were collected at different time-points. (B) Flow cytometry graphs analysis the mean fluorescent values of tripical experiments, error bars are +/- S.D.,*P<0.001; (C) HUVEC culture was maintained for 12 hours in the presence of graded concentrations of MSC-MVs. X-axis: CFSE fluorescence intensity; Y-axis: forward scatter corner signals showing the size of the gated events. (D) MVs internalization is dose-dependent (*P<0.001, n = 3).

To quantify the proportion of MV internalization, CFSE- or Dil-labeled hypoxia-induced MSC-MVs (10μg/ml) were added to the culture of HUVECs that had reached to a confluence of around 80%, and the cells were then collected at different time points for flow cytometry and confocal observation. As shown in Fig 3, DiI-labeled MVs were rapidly engulfed by HUVECs after the co-culture for two hours. The cellular accumulation of MVs continued as the duration time prolonged. The fluorescence appeared a bit sparsely at 6hr, and seemingly condensed at the time-point of 12hr. The results were supported by flow cytometric analysis. As shown in Fig 4A, the CFSE positivity increased gradually. T-test analysis proved the time-dependent increase of MV uptake by HUVECs (P<0.001, Fig 4B). Furthermore, when graded doses of MSC-MVs were kept with HUVECs for 12 hours, the percentage of CFSE-positive cells increased with the increase of MV concentrations (Fig 4C), reaching (91.33±1.12)% when MSC-MVs were added at a concentration of 20μg/ml. Statistical analysis also confirmed the dose-dependence (P<0.05, Fig 4D).

In fact, the CFSE positivity varied greatly when HUVECs from different donors were used. To standardize the experiments below, HUVECs from three subjects were selected. The observation time-point was set as 12 hours and the dose of MSC-MVs was used at 10μg/ml.

### Exogenous Anx-V greatly inhibits the internalization

The experiments above revealed that PS, CD29 and CD44 were present on the surface of the MSC-MVs (Fig 1B). These molecules have been shown to be responsible for the incorporation of MVs into host cells including endothelial cells [23–25], dendritic cells [18], tubular epithelial cells [26] and hepatocarcinoma cells [27]. Further, previous data and the results in this study have demonstrated that Anx-V is expressed on the surface of HUVECs [28]. Meanwhile, PS exists on the surface of MVs derived from a variety of cells [29–31], and the specific binding of Anx-V to PS has been long recognized [32, 33]. Therefore, it was assumed that PS, CD29 and CD44 might also attribute to the internalization of the hypoxia-induced MSC-MVs into the HUVECs.

**Fig 5:**
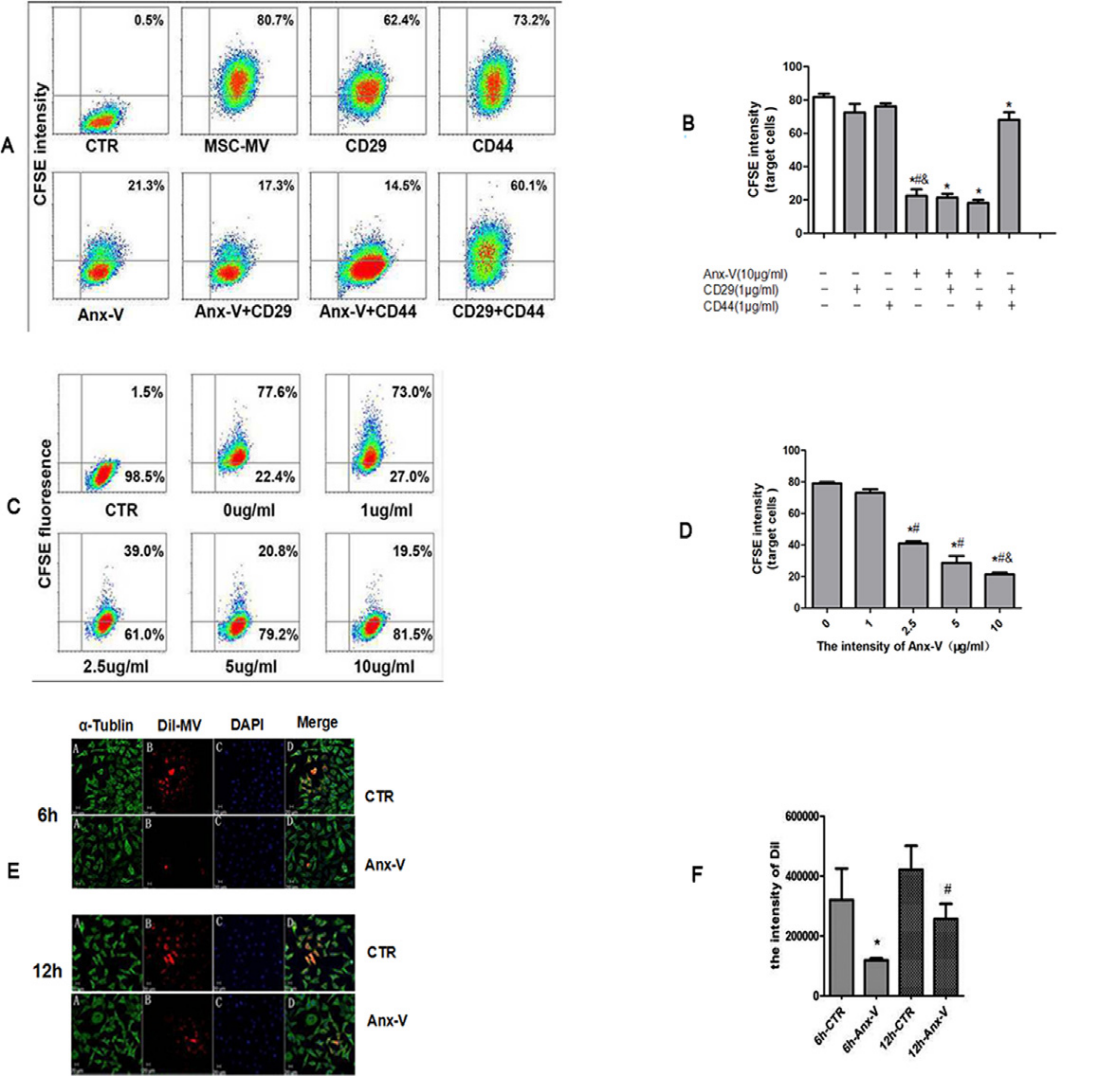
Suppression of MSC-MV incorporation into HUVECs by exogenous addition of Anx-V, anti-CD29 and anti-CD44 antibodies. (A) Flow cytometry was used to assess the CFSE intensity after the cells were cultured for 12 hours. CTR: HUVECs without MSC-MVs; MSC-MV: HUVECs with MSC-MVs only. X-axis: the forward scatter corner signals showing the cellular size. Y-axis: CFSE fluorescence intensity; (B) The percentages of the CFSE-positive cells are indicated. *P: *vs*.CTR <0.01; ^#^P: *vs*.CD29+CD44 = 0.0014; ^&^P: *vs*.CD29/CD44 <0.001. The results are representative of the data from three individual experiments. (C) Exogenous Anx-V inhibits the uptake of MSC-MVs by HUVECs in a dose-dependent manner. CTR: HUVECs in the absence of CFSE-MSC-MVs. Y-axis: CFSE intensity; X-axis: the forward scatter corner signals showing the size of the cells. (D) The Anx-V dose-dependently inhibits the MVs uptake (*P<0.001, n = 3).(E) The co-culture of HUVECs and DiI-labeled MSC-MVs was maintained in the absence (CTR) or presence of exogenous Anx-V (Anx, 10μg/ml) for 6 or 12 hours and analyzed by confocal scaning microscopy. CTR: The MSC-MVs in the absence of Anx-V. Tublin-alpha: green. DiI-MVs: red. DAPI: blue. Bar: 20μm. (F) The t-tests showed the inhibition function of Anx-V in the internalization(*vs*.6h-CTR*P<0.01;*vs*.12h-CTR^#^P<0.01).The results are representative of two separate experiments.

To testify this assumption, CFSE-labeled hypoxia-induced MSC-MVs (10μg/ml) were pretreated with recombinant human Anx-V (10μg/ml), and/or antibodies against CD29 and CD44 (1μg/ml), followed by the addition into HUVEC culture. Twelve hours later, the cells were collected for flow cytometric analysis (Fig 5A). The results showed that Anx-V dramatically blocked the incorporation of MSC-MVs (P<0.01) and its inhibition seemed not additionally augmented by the supplement of anti-CD29 or anti-CD44 antibody (P = 0.1). Meanwhile, the addition of CD29 antibody reduced the percentage of CFSE-positive cells to a greatly less extent than Anx-V did (P<0.01) and, the supplement of CD44 antibody exhibited no effect on MSC-MVs internalization (P = 0.5). The results demonstrate that the PS sites on MSC-MVs rather than CD29 and CD44 play a critical role in the incorporation process (Fig 5B).

To further confirm the involvement of PS, graded concentrations of recombinant Anx-V were added together with CFSE-labeled MSC-MVs (10μg/ml) into the culture of HUVECs. Flow cytometric analysis showed that exogenous Anx-V inhibited the uptake of MSC-MVs by HUVECs in a dose-dependent manner (Fig 5C). As shown in Fig 5D, the percentage of CFSE-positive cells decreased a bit when 1μg/ml of Anx-V was added ((73.14±3.84)%, P = 0.06), and the positivity decreased to (21.40±1.70) % when the dose increased to 10μg/ml. The inhibitory effect of Anx-V was not further augmented even when 10μg/ml of Anx-V was used. The inhibition was also identified by confocal laser microscopy (Fig 5E). And the Anx-V inhibition was apparently dose-dependent (Fig 5F).

### Blockage to PS on the MVs abrogates their ability to promote tube formation of HUVECs

**Fig 6:**
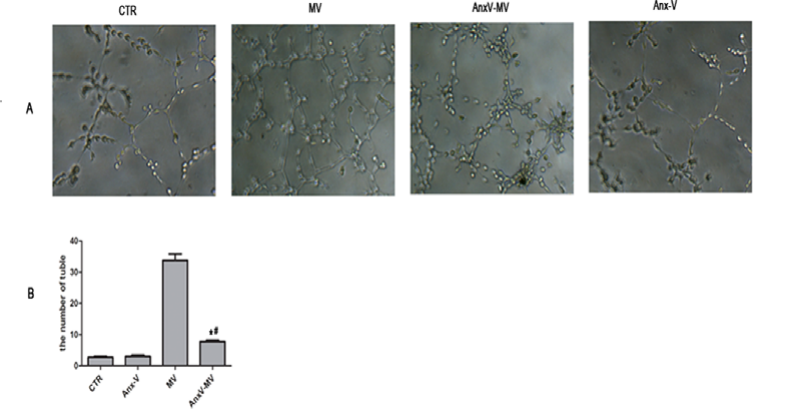
Blockage to PS on the MVs abrogates their ability to promote tube formation of HUVECs. (A) The vessel structure formation of HUVECs. CTR: Control, without MSC-MVs; MV: HUVEC+MSC-MVs; Anx-V-MV: HUVEC+Anx-V-MVs; (B) The mean number of 5 sites vessel structure (*P:*vs*.CTR <0.001;^#^P: *vs*.MVs <0.001; n = 3).

The data above suggest that PS on the surface of MVs is responsible for the internalization. It has been previously proven that MSC-MVs can greatly enhance the capacity of HUVECs to form capillary-like network. Therefore, to observe if PS blockage could affect the tube formation promoting activity, MVs mixed with 10μg/ml Anx-V protein and the mixture was co-cultured with HUVECs. The network structure was counted at the time point of 24 h. As expected, the addition of MVs significantly enhanced the number of tube-like structure compared with HUVECs without MSC-MVs (34.25±4.03 *vs*. 2.50±0.57, P<0.0001). Meanwhile, pre-treatment of MVs with Anx-V nearly totally abrogated their enhancing effect (7.75±0.95 *vs*.34.25±4.03, P < .0.0001; Fig 6).

### Blockage of Annexin-V expression with siRNA has no effect on the internalization

**Fig 7:**
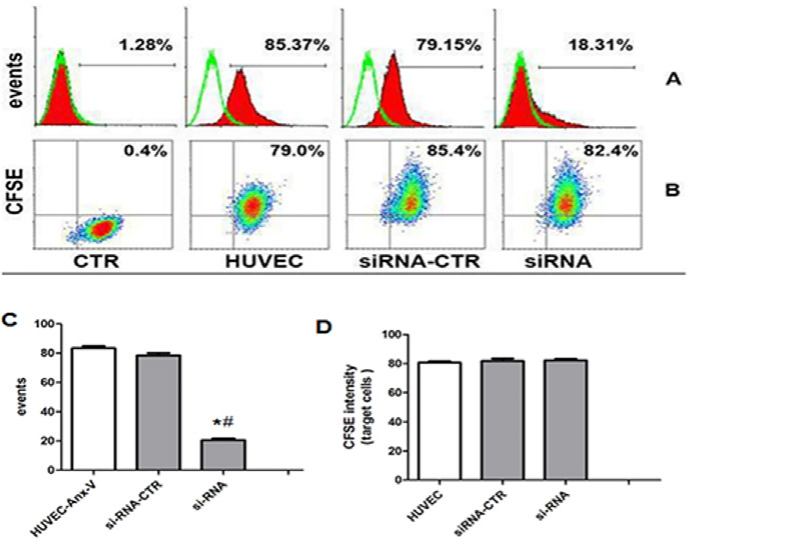
Down-regulation of Anx-V in HUVECs has little effect on the MSC-MV internalization. (A) Anx-V siRNAs were transfected into HUVECs and the Anx-V expression was assessed by flow cytometry. The hollow diagrams represent the control and the solid ones represent the Anx-V-FITC fluorescence intensities of the indicated cells. (B) HUVECs, Anx-V siRNA-transfected HUVECs (siRNA) and control siRNA-transfected HUVECs (siRNA-CTR) were maintained in culture for 12 hours in the presence of CFSE-labeled MSC-MVs. The cells were harvested for flow cytometric analysis. (C) The t-test shows that the Anx-V expression was significantly decreased by si-RNA (*vs*.CTR *P<0.001; *vs*.siRNA-CTR ^#^P<0.001). (D) Anx-V did not effect the internalization of MVs (*vs*.CTR P = 0.83). These results are representative of the data from three separate experiments.

The results above suggest that PS-Anx-V interaction might attribute to the uptaking process. To identify the possibility, siRNA for human Anx-V were transfected into HUVECs and after 72 hours, the process of MSC-MV internalization was re-assessed. The results showed that siRNA transfection could greatly inhibited Anx-V expression on HUVECs (Fig 7A). Flow cytometric analysis showed that the expression of Anx-V on HUVEC was significantly reduced by si-RNA transfection (Fig 7C; si-RNA (20.55±2.13)% *vs*.CTR (83.39±2.76%), p<0.0001; *vs*. siRNA-CTR (78.45±2.87)%, P<0.0001; n = 3). However, the down-regulation of Anx-V did not affect the incorporation of MSC-MVs into HUVECs (Fig 7B). The mean percentage of CFSE-positive cells in Anx-V siRNA-transfected HUVECs was (82.25±1.73)%, which was comparable to those of non-silencing siRNA-transfected HUVECs ((80.4±2.7)%,P = 0.83) and the counterpart cells ((80.67±1.76)%, P = 0.33) (Fig 7D). The results suggest that Anx-V on HUVECs seems not be attributed to the MVs incorporation.

### Blockage of PSR on HUVECs inhibited the MSC-MV incorporation

**Fig 8:**
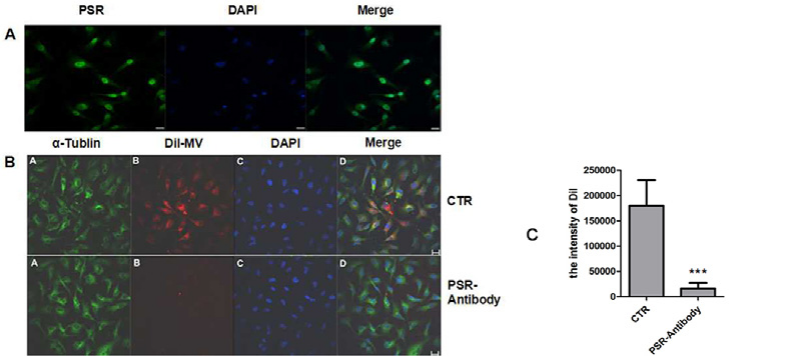
Blockage of PSR suppresses the engulfment of MSC-MVs by HUVECs. (A) PSR expression on the HUVECs was detected with confocal microscopy analysis. PSR: green; DAPI: the nuclear (blue); Bar: 20μm. (B) HUVECs were pre-treated with anti-PSR antibody and cultured in the presence of DiI-labeled MSC-MVs. Twelve hours later, the cells were fixed and observed under a confocal microscope. CTR: HUVEC with DiI-MV; PSR-Antibody: HUVEC+PSR-Antibody with DiI-MV; Bar: 20μm. (C) Blockage of PSR with a specific antibody greatly decreased the internalization of MVs into HUVECs (*vs*.CTR ^***^P<0.0001, n = 2).

The data above preclude the possibility of the contribution of PS-Anx-V interaction to the internalization process. Meanwhile, the results also suggest the critical role of PS in the MSC-MV up-taking by HUVECs. Therefore, it could be deduced that some other PS receptors on HUVECs might be responsible for this process. Previous data have demonstrated that exosomes derived from T cells could be engulfed by monocytes via the PS-PSR pathway [34]. To observe if it was the case in the interaction of hypoxia-induced MSC-MVs and HUVECs, the expression of PSR on HUVECs was evaluated with confocal microscopy. The results showed that HUVECs were homogeneously positive for PSR (Fig 8A). To assess if the PS-PSR reaction is involved in the internalization, HUVECs were pre-treated with 100μg/ml PSR antibody and then DiI-labeled MSC-MVs were co-cultured for 12 hours. The incorporation of MVs was evaluated by confocal microscopy. The results showed that the pretreatment dramatically inhibited the DiI-labeled MVs incorporation into the HUVECs. The mean fluorescence intensity per field in PSR antibody-treated HUVECs was 16264±19402, which was significantly lower than that of untreated HUVECs (180030±87314, P = 0.038, Fig 8B).

**Fig 9:**
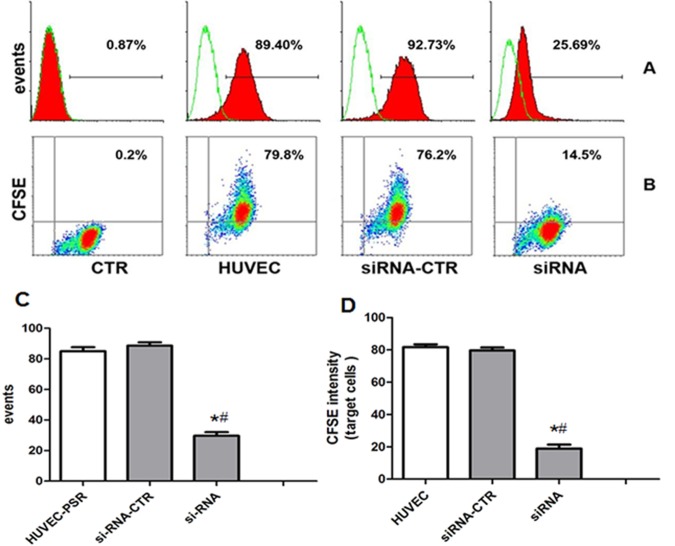
Down-regulation of PSR in HUVECs inhibits their up-taking of MSC-MVs. (A) PSR expression on HUVECs, PSR siRNA-transfected HUVECs (siRNA) and control RNA-treated HUVECs (siRNA-CTR) was evaluated by flow cytometry. The hollow diagrams represent the fluorescence intensity of cells reacted with the FITC-conjugated antibody. (B) The uptake of CFSE-labeled MSC-MVs was observed with flow cytometry. X-axis: the forward scatter corner signals. Y-axis: CFSE intensity. (C)The t-test showed that the PSR expression was significantly decreased by si-RNA (vs.CTR * P<0.05; vs.siRNA-CTR # P<0.05). (D) PSR siRNA significantly inhibited the internalization of MVs (vs.CTR * P<0.05; vs.siRNA-CTR # P<0.05). These results are representative of the data from three separate experiments.

To futher observe if it was the case in the interaction of the hypoxia-induced MSC-MVs and HUVECs, the expression of PSR on HUVECs was also evaluated by flow cytometry. The results showed that HUVECs were positive for PSR, and PSR siRNA specifically down-regulated its expression (Fig 9A). After incubation with CFSE-labeled MSC-MVs for 12 hours, most of the PSR siRNA-transfected HUVECs were negative for CFSE, in sharp contrast to non-silencing RNA-transfected HUVECs and their counterparts (Fig 9B). The mean CFSE-positive percentage from three experiments was (85.02±4.47)% in HUVECs, and that in control siRNA-transfected cells was (78.45±2.87)%. Meanwhile, the positive percentage was greatly reduced in PSR siRNA-treated HUVECs ((18.97±4.18) %,P<0.05) (Fig 9B).

## Discussion

In the present study, we have intended to clarify the process of incorporation hypoxia-induced MSC-MVs into HUVECs and found that blockage of PS on the surface of MSC-MVs with exogenous Anx-V other than antibodies against CD29 and CD44 greatly inhibits the internalization. Furthermore, the blockage of the PS on MVs with exogenous Anx-V diminished the effect of tube formation-promting MSC-MVs. However, down-regulation of Anx-V expression with si-RNA technique has little/no effect on this process. And interestingly, the addition of anti-PSR antibody greatly blocked the incorporation of the MVs into HUVECs. The results here support the conclusion that PS is a critical molecule in the up-taking of hypoxia-induced MSC-MVs by HUVECs, and probably via the interaction of PS on the MSC-MVs and PSR on the HUVECs, though the detailed mechanisms underlying the interaction and the subsequent signaling remain still unknown. However, it should be noted that the PS-PSR pathway may not be the exclusive one responsible for the cellular incorporation of MSCs-MVs, as blockage of PS on MVs with exogenous Anx-V and PSR on HUVECs could not completely shut off the entrance of MSC-MVs into the endothelial cells.

Generally, MVs are originated through different mechanisms, including the apoptotic bodies from dying cells, plasma membrane blebbing (ectosomes) and the endosomal processing and emission of plasma membrane (exosomes) from activated and apoptotic cells [35]. In the present study, MVs were collected from the supernatants of MSCs that had exposed to hypoxic and serum-deprivation. Electron microscopy showed that the MVs were round- or cup-shaped and less than 100nm in diameter. Further, most of the MVs were positive for PS. Thus, the MSC-MVs described here might be a mixture of ectosomes and exosomes without the contamination of apoptotic bodies.

PS exposure is a prominent feature of MVs and this phenomenon has been discovered in MVs derived from platelets, red cells, multiple myeloma cells, melanocyts and other cell types [29, 36, 37]. PS has been proven to take a critical part in the internalization of MVs into endothelial cells, in which the MVs were originated from a variety of cells including platelets, monocytes, squamous cell carcinoma cells, and endothelial cells [18,26,27]. PS is also involved in the uptake of extracellular exosomes by dendritic cells [25]. In this study, we have found that blockage of PS sites on hypoxia-induced MSC-MVs with Anx-V greatly reduces the incorporation into HUVECs, though the inhibition is incomplete. The results demonstrate that PS on hypoxia-induced MSC-MVs is a key molecule responsible for the internalization and, other surface molecules might also play a role in the process. A previous report has found that CD44 and CD29, two marker molecules expressed on MSCs and MSC-MVs, are involved in the incorporation of MSC-MVs into kidney tubular epithelial cells [26]. Further, the incorporation of liver stem cell-derived MVs into HepG2 hepatoma cells appears to be CD29 dependent [27]. However, our data here show that Anx-V could greatly suppress other than completely blocking MSC-MV incorporation, while the addition of monoclonal antibodies against CD44 and CD29 had no effect on the uptake of MSC-MVs by HUVECs, and Anx-V supplemented with anti-CD29 antibody enhanced the inhibitory effect of Anx-V only to a little extent (Fig 4). The inconsistency might hint that the varieties of the host cells, the cellular origins of MVs and the stimuli that the cells have received might result in the differences of the pathway(s) the cells will take to engulf MVs, as the MV internalization is an active reaction of the host cells rather than merely casual attachment of MVs to the cell surface [38, 39]. The MVs isolated from non hypoxic cells no PS exposure on their surface, however, they could be internalized by HUVECs, and the results was conformity with our previous data. The results showed in the S2 Fig so we cannot exclude the possibilities that other pathways such as lactadherin/MFG-E8, integrins and endocytosis might be involved in the internalization of MVs derived from routinely cultured MSCs or cells exposed to hypoxic. A recent study has shown that MVs from several tumor cell lines entered into the host cells mainly via the cell-surface heparan sulfate proteoglycans and, blockage to these proteoglycans nearly completely intercepted the MV internalization [40]. The data suggest that CD29 and other members of the integrin family are critical players in the indicated settings. Therefore, further investigations are needed to discover the molecules that might assist or provide supplementary activity on the functionality of PS on MSC-MVs.

PS, the most well-studied marker of cells apoptosis, is translocated to the outer leaflet of plasma membrane, and can bind specially to a variety of surface receptors on the cells including phagocytes, epithelial cells, and endothelial cells. These receptors such as PSR, C. Elegan CED-1, the tyrosine kinase receptor MER, integrins and TIM family members, can recognize and link the PS directly or indrectly [41–45]. Anx-V, a three-dimensional structure that is a non-glycoaylated single chain protein composed of 319 amino acid residues with a molecular mass of 35.7kDa, was first described functionally as a vascular anticoguant. Now Anx-V is considered to be the most common candidate molecules to detect the PS on the membrane of the apoptotic cells, though the underlying function of Anv-V is still unclear [46–47]. However, inhibition of Anx-V expression on HUVECs by si-RNA targeting has no effect on the internalization of MVs into HUVECs. The results suggest that other PS ligands other than Anx-V might be attributed to the incorporation.

PSR is an well-known classic receptor for PS. Furthermore, the tethering and ingestion of erythrocyte apoptotic bodies by vascular smooth muscle cells depend on the interaction of PS and PSR [48]. Additionally, Tim-1 and Tim-4 are needed in the PS-mediated clearance of apoptotic bodies by phagocytes [49,50]. Recent studies have demonstrated that the internalization of MVs into macrophages and tumor cells are also PS-mediated [25, 39]. However, the details about the incorporation of MVs into endothelial cells are still unknown [24, 51, 52]. In the present study, we have demonstrated that specific inhibition of Anx-V expression on HUVECs by the interference siRNAs could not suppress the MSC-MV incorporation, while the uptake was significantly restrained in PSR siRNA-transfected or PSR antibody treated HUVECs. The results suggest that PS-PSR bridging plays a critical role in the incorporation of hypoxia-induced MSC-MVs into HUVECs. The phenomenon has also been observed in the clearance of apoptotic bodies by phagocytes and endothelial cells [39, 41, 50, 53]. However, PSR blockage could not thoroughly shut off the entrance of MSC-MVs into HUVECs, hinting that some other pathways might also be involved in the process. Furthermore, the mechanisms underlying the PS-PSR bridging and the subsequent intracellular signaling activities should be clarified with more detailed investigations.

In conclusion, the present study provides definite evidences to support the view-point that PS on the surface of hypoxia-induced MSC-MVs and PSR on HUVECs are important participants in the internalization of hypoxia-induced MSC-derived MVs into HUVECs. Previous data have confirmed that the angiogenesis-promoting activities of MSCs are partially due to the interaction of the MSC-MVs with endothelial cells [11–13]. The discovery of the details underlying the interaction might provide novel strategies to design therapeutic measures in the management of cardiovascular and peripheral blood vessel diseases.

## Supporting Information

S1 Fig MVs derived from non hypoxic cells were conjugated with aldehyde/sulfate latex beads and reacted with Anx-V-FITC. The events were collected with a flow cytometer and the single beads (green) and the doublets of beads (red) were gated for further analysis. The percentages of the positivity in contrast to an isotype antibody are indicated. X-axis: forward scatter corner signals showing the size of the gated events. Beads: Beads were collected for the determination of the gates. Beads+MVs: MVs conjugated with beads were collected for further determination of the gates for analysis. CTR: Beads reacted with Anx-V-FITC.
(TIF)

S2 Fig The uptake of MVs isolated from non hypoxic cells by HUVECs. The hollow diagrams represent the control and the solid ones represent the CFSE fluorescence intensities of the indicated cells. X-axis: the relative fluorescence of CFSE, and Y-axis: the number of events; CTR: Control, without MSC-MVs; MV: HUVEC+CFSE-MSC-MVs(10μg/ml).
(TIF)

## Abbreviations

MVs: Microvesicles
CFSE: Carboxyfluorescein Succinimidyl Ester
UC: Umbilical cord
MSC: mesenchymal stem cells
ECs: endothelial cells
FBS: fetal bovine serum
PE: phycoerythrin
FITC: fluorescent isothiocyanate
PS: phosphatidylserine
PSR: phosphatidylserine receptor
Anx-V: annexin-V

## References

[1] Lai Ruenn Chai, Yeo Ronne Wee Yeh, Tan Kok Hian & Lim Sai Kiang. (2013) Mesenchymal stem cell exosome ameliorates reperfusion injury through proteomic complementation. Regen Med. 8(2):197–209. 10.2217/rme.13.4 . 23477399

[2] Rosado-de-Castro PH, Pimentel-Coelho PM, da Fonseca LM, de Freitas GR, Mendez-Otero R, Rosado-de-Castro Paulo Henrique, et al (2013) The rise of cell therapy trials for stroke: review of published and registered studies. Stem Cells Dev. 22(15):2095–111. 10.1089/scd.2013.0089 . 23509917 PMC3715770

[3] Watt SM, Gullo F, van der Garde M, Markeson D, Camicia R, Khoo CP, et al (2013) The angiogenic properties of mesenchymal stem/stromal cells and their therapeutic potential. Br Med Bull. 2013;108:25–53. 10.1093/bmb/ldt031 . 24152971 PMC3842875

[4] Caplan AI. (2009) Why are MSCs therapeutic? New data: new insight. J Pathol. 217(2):318–24. 10.1002/path.2469 . 19023885 PMC8793150

[5] Kinnaird T, Stabile E, Burnett MS, Epstein SE. (2004) Bone-marrow-derived cells for enhancing collateral development: mechanisms, animal data, and initial clinical experiences. Circ Res 95:354–363. . 15321945 10.1161/01.RES.0000137878.26174.66

[6] Lasala GP, Silva JA, Gardner PA, Minguell JJ. (2010) Combination stem cell therapy for the treatment of severe limb ischemia: safety and efficacy analysis. Angiology. 61(6):551–6. 10.1177/0003319710364213 . 20498146

[7] Caplan AI, Correa D. (2011) The MSC: an injury drugstore. Cell Stem Cell. 9(1):11–5. 10.1016/j.stem.2011.06.008 21726829 PMC3144500

[8] Qayyum AA, Haack-Sørensen M, Mathiasen AB, Jørgensen E, Ekblond A, Kastrup J. (2012) Adipose-derived mesenchymal stromal cells for chronic myocardial ischemia (MyStromalCell Trial): study design. Regen Med.7(3):421–8. 10.2217/rme.12.17 . 22594332

[9] Smith RR, Barile L, Messina E, Marbán E. (2008) Stem cells in the heart: what's the buzz all about? Part 2: Arrhythmic risks and clinical studies. Heart Rhythm. 5(6):880–7. 10.1016/j.hrthm.2008.02.011 . 18534373 PMC2717007

[10] Minguell JJ, Allers C, Lasala GP. (2013) Mesenchymal stem cells and the treatment of conditions and diseases: the less glittering side of a conspicuous stem cell for basic research. Stem Cells Dev. 22(2):193–203. 10.1089/scd.2012.0417 . 23025629

[11] Zhang HC, Liu XB, Huang S, Bi XY, Wang HX, Xie LX, et al (2012) Microvesicles derived from human umbilical cord mesenchymal stem cells stimulated by hypoxia promote angiogenesis both in vitro and in vivo. Stem Cells Dev.21(18):3289–97. 10.1089/scd.2012.0095 . 22839741 PMC3516422

[12] Bian S, Zhang L, Duan L, Wang X, Min Y, Yu H. (2014) Extracellular vesicles derived from human bone marrow mesenchymal stem cells promote angiogenesis in a rat myocardial infarction model. J Mol Med (Berl). 92(4):387–97. 10.1007/s00109-013-1110-5 24337504

[13] Lopatina T, Bruno S, Tetta C, Kalinina N, Porta M, Camussi G. (2014) Platelet-derived growth factor regulates the secretion of extracellular vesicles by adipose mesenchymal stem cells and enhances their angiogenic potential. Cell Commun Signal. 12:26 10.1186/1478-811X-12-26 . 24725987 PMC4022079

[14] Escrevente Cristina, Keller Sascha, Altevogt Peter and Costa Júlia. (2011) Interaction and uptake of exosomes by ovarian cancer cells. Escrevente et al BMC Cancer.11:108 10.1186/1471-2407-11-108 . 21439085 PMC3072949

[15] Svensson Katrin J., Christianson Helena C., Wittrup Anders, Erika. (2013) Exosome uptake depends on ERK1/2-heat shock protein 27 signalling and lipid raft-mediated endocytosis negatively regulated by caveolin-1. J Biol Chem. 2013 6 14;288(24):17713–24. 10.1074/jbc.M112.445403 . 23653359 PMC3682571

[16] Feng Du, Zhao Wen-Long, Ye Yun-Ying, Bai Xiao-Chen, Liu Rui-Qin, Chang Lei-Fu, et al (2011) Cellular Internalization of Exosomes Occurs Through Phagocytosis [J]. Traffic.11(5):675–87. 10.1111/j.1600-0854.2010.01041.x . 20136776

[17] Janko Christina, Jeremic Ivica, Biermann Mona, Chaurio R, Schorn C, Muñoz LE, et al (2013) Cooperative binding of Annexin A5 to phosphatidylserine on apoptotic cell membranes [J]. Phys Biol. 10(6):065006 10.1088/1478-3975/10/6/065006 . 24304966

[18] Morelli AE, Larregina AT, Shufesky WJ, Sullivan ML, Stolz DB, Papworth GD, et al (2004) Endocytosis, intracellular sorting, and processing of exosomes by dendritic cells [J]. Blood.104(10):3257–66. . 15284116 10.1182/blood-2004-03-0824

[19] Keller S, König AK, Marmé F, Runz S, Wolterink S, Koensgen D, et al (2009) Systemic presence and tumor-growth promoting effect of ovarian carcinoma released exosomes.Cancer Lett. 6 8;278(1):73–81. 10.1016/j.canlet.2008.12.028 . 19188015

[20] Jin JD, Wang HX, Xiao FJ, Wang JS, Lou X, Hu LD, et al (2008) A novel rich source of human mesenchymal stem cells from the debris of bone marrow samples. Biochem Biophys Res Commun 376(1):191–195. 10.1016/j.bbrc.2008.08.131 . 18774774

[21] Dominici M, Le Blanc K, Mueller I, Slaper-Cortenbach I, Marini F, Krause D, et al (2006) Minimal criteria for defining multipotent mesenchymal stromal cells. The International Society for Cellular Therapy position statement. Cytotherapy 8:315–317. . 16923606 10.1080/14653240600855905

[22] Wehder L, Arndt S, Murzik U, Bosserhoff AK, Kob R, von Eggeling F, et al (2009) Annexin A5 is involved in migration and invasion of oral carcinoma. Cell Cycle 8:1552–1558. . 19372761 10.4161/cc.8.10.8404

[23] Al-Nedawi K, Meehan B, Kerbel RS, Allison AC, Rak J. (2009) Endothelial expression of autocrine VEGF upon the uptake of tumor-derived microvesicles containing oncogenic EGFR. Proc Natl Acad Sci U S A 106(10):3794–3799. 10.1073/pnas.0804543106 . 19234131 PMC2656159

[24] Terrisse AD, Puech N, Allart S, Gourdy P, Xuereb JM, Payrastre B, et al (2010) Internalization of microparticles by endothelial cells promotes platelet/endothelial cell interaction under flow. J Thromb Haemost 8(12): 2810–9. 10.1111/j.1538-7836.2010.04088.x . 21029362

[25] Lima LG, Leal AC, Vargas G, Porto-Carreiro I, Monteiro RQ. (2013) Intercellular transfer of tissue factor via the uptake of tumor-derived microvesicles. Thromb Res 132(4):450–456. 10.1016/j.thromres.2013.07.026 . 23993901

[26] Bruno S, Grange C, Deregibus MC, Calogero RA, Saviozzi S, Collino F, et al (2009) Mesenchymal stem cell-derived microvesicles protect against acute tubular injury. J Am Soc Nephrol 20(5):1053–1067. 10.1681/ASN.2008070798 . 19389847 PMC2676194

[27] Fonsato V, Collino F, Herrera MB, Cavallari C, Deregibus MC, Cisterna B, et al (2012) Human liver stem cell-derived microvesicles inhibit hepatoma growth in SCID mice by delivering antitumor microRNAs. Stem Cells 30(9):1985–1998. 10.1002/stem.1161 . 22736596 PMC3468738

[28] Wang X, Campos B, Kaetzel MA, Dedman JR. (2001) Secretion of annexin V from cultured cells requires a signal peptide. Placenta 22:837–845. . 11718571 10.1053/plac.2001.0724

[29] Jayachandran M, Miller VM, Heit JA, Owen WG. (2012) Methodology for isolation, identification and characterization of microvesicles in peripheral blood. J Immunol Methods 375(1–2):207–214. 10.1016/j.jim.2011.10.012 . 22075275 PMC3253871

[30] Qu J, Adam J, Bloxham DM, Bruckdorfer KR, Miller NG, Parkinson NA, et al (2000) Phosphatidylserine-dependent adhesion of T cells to endothelial cells. Biochim Biophys Acta 1501:99–115. 10838184 10.1016/s0925-4439(00)00022-3

[31] Xiong J, Miller VM, Li Y, Jayachandran M. (2012) Microvesicles at the crossroads between infection and cardiovascular diseases. J Cardiovasc Pharmacol 59(2):124–132. 10.1097/FJC.0b013e31820c6254 . 21242813 PMC3090703

[32] Thiagarajan P, Tait JF. (1990) Binding of annexin V/placental anticoagulant protein I to platelets. Evidence for phosphatidylserine exposure in the procoagulant response of activated platelets. J Biol Chem.265(29):17420–3. . 2145274

[33] Ravanat C, Archipoff G, Beretz A, Freund G, Cazenave JP, Freyssinet JM. (1992) Use of annexin-V to demonstrate the role of phosphatidylserine exposure in the maintenance of haemostatic balance by endothelial cells. Biochem J 282 (Pt 1):7–13. . 1311563 10.1042/bj2820007 PMC1130883

[34] Zakharova L, Svetlova M, Fomina AF. (2007) T cell exosomes induce cholesterol accumulation in human monocytes via phosphatidylserine receptor. J Cell Physiol 212(1):174–181. . 17299798 10.1002/jcp.21013

[35] Lee TH, D'Asti E, Magnus N, Al-Nedawi K, Meehan B, Rak J. (2011) Microvesicles as mediators of intercellular communication in cancer—the emerging science of cellular 'debris'. Semin Immunopathol 33(5):455–467. 10.1007/s00281-011-0250-3 . 21318413

[36] Liu Y, Zhu XJ, Zeng C, Wu PH, Wang HX, Chen ZC, et al (2014) Microvesicles secreted from human multiple myeloma cells promote angiogenesis. Acta Pharmacol Sin 35(2):230–238. 10.1038/aps.2013.141 . 24374814 PMC4651219

[37] György B, Pálóczi K, Kovács A, Barabás E, Bekő G, Várnai K, et al (2014) Improved circulating microparticle analysis in acid-citrate dextrose (ACD) anticoagulant tube. Thromb Res 133(2):285–292. 10.1016/j.thromres.2013.11.010 . 24360116

[38] Lima LG, Oliveira AS, Campos LC, Bonamino M, Chammas R, Werneck C, et al (2011) Malignant transformation in melanocytes is associated with increased production of procoagulant microvesicles. Thromb Haemost 106(4):712–723. 10.1160/TH11-03-0143 . 21800005

[39] Frey B, Gaipl US. (2011) The immune functions of phosphatidylserine in membranes of dying cells and microvesicles. Semin Immunopathol 33(5):497–516. 10.1007/s00281-010-0228-6 . 20941495

[40] Christianson HC, Svensson KJ, van Kuppevelt TH, Li JP, Belting M. (2013) Cancer cell exosomes depend on cell-surface heparan sulfate proteoglycans for their internalization and functional activity. Proc Natl Acad Sci U S A 110(43): 17380–17385. 10.1073/pnas.1304266110 . 24101524 PMC3808637

[41] Setty BN, Betal SG. (2008) Microvascular endothelial cells express a phosphatidylserine receptor: a functionally active receptor for phosphatidylserine-positive erythrocytes. Blood 111:905–914. . 17911385 10.1182/blood-2007-07-099465 PMC2200830

[42] Shang Y, Li Z, Li H, Xia H, Lin Z.(2013) TIM-3 expression in human osteosarcoma: Correlation with the expression of epithelial-mesenchymal transition-specific biomarkers. Oncol Lett 6(2):490–494. . 24137353 10.3892/ol.2013.1410 PMC3789023

[43] Li J, Cao D, Guo G, Wu Y, Chen Y. (2013) Expression and anatomical distribution of TIM-containing molecules in Langerhans cell sarcoma. J Mol Histo 44(2): 213–220. 10.1007/s10735-012-9475-2 . 23264111

[44] Lauber Kirsten, Blumenthal Sibylle G., Waibel Michaela, Wesselborg Sebastian. (2004) Clearance of Apoptotic Cells: Getting Rid of the Corpses. Molecular Cell. 14(3): 277–287. . 15125832 10.1016/s1097-2765(04)00237-0

[45] Arur Swathi, Uche Uche E., Rezau Karim, Fong Michael, Scranton Victoria, Cowan Ann E., et al (2003) Annexin I Is an Endogenous Ligand that Mediates Apoptotic Cell Engulfment. Developmental Cell, 4(4): 587–598. . 12689596 10.1016/s1534-5807(03)00090-x

[46] Penga Boya, Guoa Chunmei, HongweiGuanb, ShuqingLiuc, Ming-ZhongSun. (2014) Annexin A5 as a potential marker in tumors. Clinica Chimica Acta, 427: 42–48. 10.1016/j.cca.2013.09.048 . 24121031

[47] Shena Hsin-Hui, Lakec Vanessa, Le Brunc Anton P., Jamesd Michael, Duffc Anthony P., Penga Yong, et al (2013) Targeted detection of phosphatidylserine in biomimetic membranes and in vitro cell systems using annexin V-containing cubosomes. Biomaterials, 34(33): 8361–8369. 10.1016/j.biomaterials.2013.07.042 . 23899446

[48] Kolb S, Vranckx R, Huisse MG, Michel JB, Meilhac O. (2007) The phosphatidylserine receptor mediates phagocytosis by vascular smooth muscle cells. J Pathol 212(3): 249–259. . 17534843 10.1002/path.2190

[49] Kobayashi N, Karisola P, Peña-Cruz V, Dorfman DM, Jinushi M, Umetsu SE, et al (2007) TIM-1 and TIM-4 glycoproteins bind phosphatidylserine and mediate uptake of apoptotic cells. Immunity 27(6):927–940. . 18082433 10.1016/j.immuni.2007.11.011 PMC2757006

[50] Zhou Z. (2007) New phosphatidylserine receptors: clearance of apoptotic cells and more. Dev Cell 13(6):759–760. . 18061557 10.1016/j.devcel.2007.11.009

[51] Soleti R, Lauret E, Andriantsitohaina R, Carmen Martínez M. (2012) Internalization and induction of antioxidant messages by microvesicles contribute to the antiapoptotic effects on human endothelial cells. Free Radic Biol Med 53(11): 2159–2170. 10.1016/j.freeradbiomed.2012.09.021 . 23010499

[52] Deregibus MC, Cantaluppi V, Calogero R, Lo Iacono M, Tetta C, Biancone L, et al (2007) Endothelial progenitor cell derived microvesicles activate an angiogenic program in endothelial cells by a horizontal transfer of mRNA. Blood 110(7): 2440–2448. . 17536014 10.1182/blood-2007-03-078709

[53] Hoffmann PR, deCathelineau AM, Ogden CA, Leverrier Y, Bratton DL, Daleke DL, et al (2001) Phosphatidylserine (PS) induces PS receptor-mediated macropinocytosis and promotes clearance of apoptotic cells. J Cell Biol 155(4):649–659. . 11706053 10.1083/jcb.200108080 PMC2198875

