# Supplementary figures and images for: Surface Phosphatidylserine Is Responsible for the Internalization on Microvesicles Derived from Hypoxia-Induced Human Bone Marrow Mesenchymal Stem Cells into Human Endothelial Cells
Source: PLoS One. 2016 Jan 25;11(1):e0147360. doi: 10.1371/journal.pone.0147360 (PMC4726621; doi:10.1371/journal.pone.0147360)

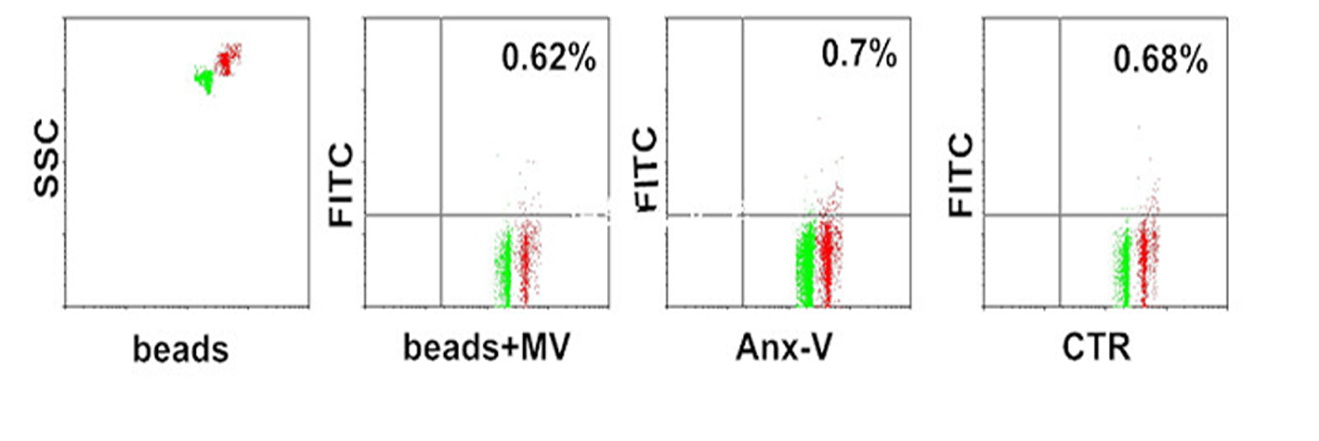

Supplement: S1 Fig — The events were collected with a flow cytometer and the single beads (green) and the doublets of beads (red) were gated for further analysis. The percentages of the positivity in contrast to an isotype antibody are indicated. X-axis: forward scatter corner signals showing the size of the gated events. Beads: Beads were collected for the determination of the gates. Beads+MVs: MVs conjugated with beads were collected for further determination of the gates for analysis. CTR: Beads reacted with Anx-V-FITC. (TIF) [file pone.0147360.s001.tif]

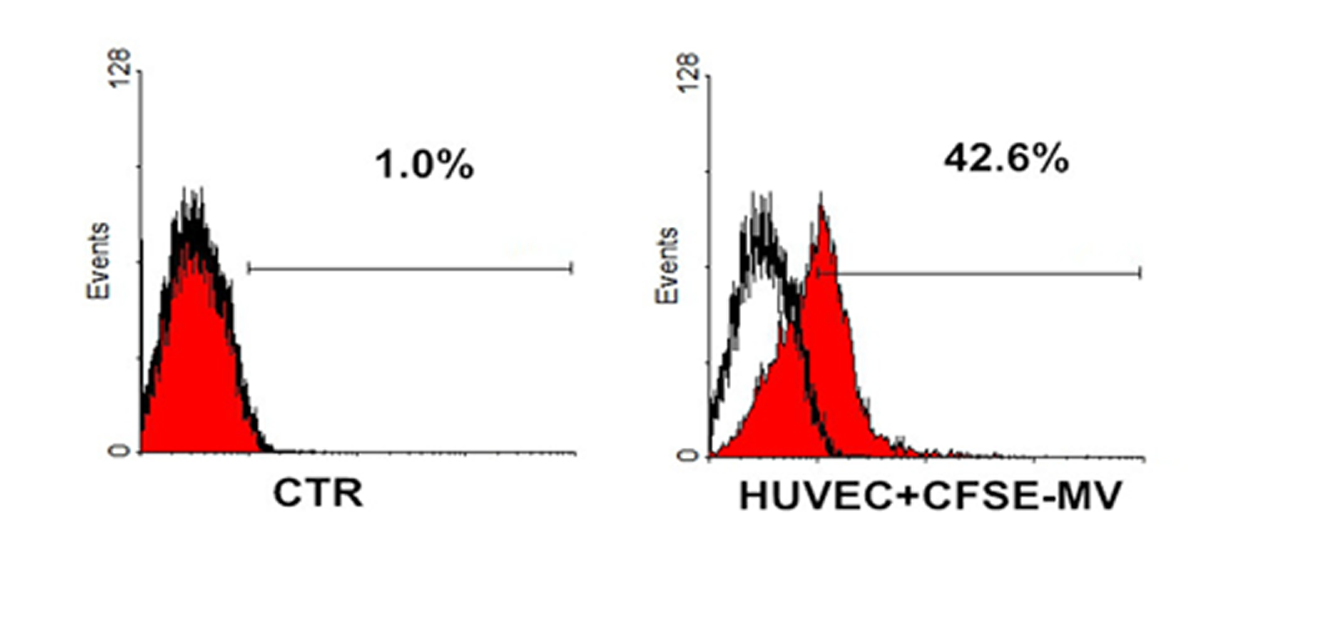

Supplement: S2 Fig — The hollow diagrams represent the control and the solid ones represent the CFSE fluorescence intensities of the indicated cells. X-axis: the relative fluorescence of CFSE, and Y-axis: the number of events; CTR: Control, without MSC-MVs; MV: HUVEC+CFSE-MSC-MVs(10μg/ml). (TIF) [file pone.0147360.s002.tif]
